# T cell immuno-phenotyping : a source of predictive biomarkers for autoimmune hepatitis relapse

**DOI:** 10.1038/s41598-024-75624-6

**Published:** 2024-10-18

**Authors:** Astrid Imbert, Pierre-Jean Gavlovsky, Jean-Paul Judor, Edouard Bardou-Jacquet, Laure Elkrief, Adrien Lannes, Christine Silvain, Mathieu Schnee, Florence Tanne, Caroline Chevalier, Fabienne Vavasseur, Marion Khaldi, Sophie Brouard, Jean-François Mosnier, Jérôme Gournay, Sophie Conchon, Amédée Renand

**Affiliations:** 1 Nantes Université, CHU Nantes, INSERM, Centre de Recherche Translationnelle en Transplantation et Immunologie, UMR 1064, F-44000 Nantes, France; 2 CHU Nantes, Nantes Université, Service Hépato-Gastroentérologie, IMAD, Nantes, France; 3 CHU Nantes, INSERM, Centre d’Investigation Clinique IMAD, Nantes, France; 4 CHU Nantes, Nantes Université, Service Anatomie et Cytologie Pathologiques, Nantes, France; 5 CHU Rennes, Service des maladies du foie, Rennes, France; 6 CHRU Tours, Service Hépato-Gastroentérologie, Tours, France; 7 CHU Angers, Service Hépato-Gastroentérologie et Oncologie Digestive, Angers, France; 8 Université d’Angers, Laboratoire HIFIH, UPRES EA3859, SFR 4208, Angers, France; 9 CHU Poitiers, Service Hépato-Gastroentérologie, Poitiers, France; 10 CHD Vendée-La Roche sur Yon, Service Hépato-Gastroentérologie, F- 85000 la Roche sur Yon, France; 11 CHU Brest, Service Hépato-Gastroentérologie, Brest, France; 12 CR2TI, UMR 1064, 30 Bd Jean Monnet, 44093 Nantes, France

**Keywords:** Peripheral helper T cells, CD38, BAFF, Biomarkers, Personalized medicine, Autoimmunity, Hepatology, T cells

## Abstract

Relapse after immunosuppression (IS) treatment withdrawal is frequent in patients with Autoimmune Hepatitis (AIH), and non-invasive biomarkers predictive of this risk are lacking. We assessed the frequency of circulating T cell subsets as potential biomarkers of disease activity and predictor of the risk of relapse after IS withdrawal. Serum levels of the cytokine B-cell Activating Factor (BAFF) were also investigated. Blood samples from 58 patients with active AIH, 56 AIH patients in remission, and 31 patients with NASH were analyzed. The frequency of activated CD4+ T peripheral helper (TPH) cells (CD4+CD45RA-CXCR5-PD1+CD38+) and of activated CD8+ T cells (CD8+CD45RA-PD1+CD38+) were assessed by flow cytometry. BAFF levels were determined by ELISA. Activated TPH and CD8+ T cell frequencies were significantly increased in patients with active AIH compared to remission AIH or NASH (TPH: 0.88% of total CD3+ vs. 0.42% and 0.39% respectively, *p* < 0.0001; CD8+ subset: 1.42% vs. 0.09% and 0.11% *p* < 0.0001). Among patients in remission undergoing treatment withdrawal (*n* = 18), those with increased frequencies of activated TPH (> 0.5% of total CD3+) and/or activated CD8+ T cells (> 0.18% total CD3+) had a higher risk of relapse (80% vs. 15% after 2 years, *p* = 0.0071). High BAFF serum concentration (> 213pg/ml) was also associated to a higher risk of relapse (57% vs. 11%, *p* = 0.0452). In conclusion, high frequency of activated TPH and of activated CD8+, as well as high levels of BAFF, before IS discontinuation, were significantly associated to a greater risk of relapse during the first two years. Thus, they represent promising biomarkers to provide personalized clinical follow-up for patients with AIH.

## Introduction

Autoimmune hepatitis (AIH) is a rare disease characterized by inflammation of the liver induced by abnormal reactivity of the adaptative immune system against self-antigens. Its pathogenesis remains still partly unknown^1,2^.

Treatment of AIH relies firstly on steroids to obtain an initial response and then on long-term Immunosuppression (IS) to control the disease and prevent new flares. The objective is to achieve complete biochemical response (CBR), and histological remission (Hepatitis activity index < 4/18) and prevent fibrosis progression. Most patients require long-term immunosuppressive therapy with potential adverse effects. Treatment can be successfully discontinued in some patients, but unfortunately, relapse after drug withdrawal is frequent, ranging between 50–90%^3–5^.

To limit the risk of relapse, international guidelines recommend maintaining patients on CBR for 2 years before attempting a gradual cessation of treatment^6^. A follow-up liver biopsy could be informative when considering discontinuation of treatment, but is not recommended by current guidelines and rarely performed. Indeed, liver biopsies are poorly accepted by patients, despite their strong wish to stop their treatment^7^. Some clinical and biological criteria could aid in predicting the chance to maintain biochemical remission after drug withdrawal, however they require further confirmation and proper predictors for relapse remain to be identify^3,5,8^. Immune biomarkers as potential predictors of the risk of relapse after discontinuation of IS could arise from the better understanding of the immune pathogenesis of AIH.

The dual role of CD4 T cells is thought to be crucial in autoimmune disorders. The presentation of autoantigens by antigen presenting cells to naïve CD4+ T cells through class II HLA (Major Histocompatibility class II, MHC-II), induces their differentiation into autoreactive T cells in a MHC-II: peptide: TCR-complex-dependent manner. Autoreactive CD4+ T cells with a Th1 phenotype produce Interferon γ (IFN γ) and promote CD8+ T cell cytotoxicity, implicated in liver damage. Mature (memory) autoreactive CD4+ T cells also promote, via the production of IL21, the differentiation of B cells into memory B cells and autoreactive plasma cells which, in turn, produce autoantibodies and contribute to elevated levels of Immunoglobulin G (IgG).

Classically, the differentiation of B cells into plasma cells takes place in germinal centers and is helped by CD4+ T Follicular Helper (TFH) cells expressing CXCR5 and PD1 ^9^. Recently, autoreactive T Peripheral Helper (TPH) with a CXCR5-PD1+ profile, have also been shown to induce plasma cells, and have been described as potential mediators of autoimmunity in systemic lupus erythematosus, multiple sclerosis, type 1 diabetes and rheumatoid arthritis^10–13^.

In AIH, the role of TFH appears still unclear and controversial^14,15^. Previously, we have studied autoreactive CD4+ T cells in AIH patients, and revealed their phenotype, CXCR5-PD1+CD45RA- CD38+CD127-CD27+, close to that of the TPH cells. Based on this multiparametric analysis, a high frequency of activated (CD38+) TPH was found associated with the disease activity in a limited number of AIH patients, suggesting its potential use as a biomarker in clinical practice^16^.

Some of the diagnosis criteria of the disease, such as the elevated IgG and the presence of specific autoantibodies in the serum^17^, also suggest the involvement of B cells and autoreactive plasma cells in AIH pathogenesis. The cytokine B-cell Activating Factor (BAFF) helps the differentiation, proliferation and survival of B cells^18^ and its serum level could be associated to the course of the disease. A high level of BAFF at diagnosis has been shown to be associated with a more severe presentation (higher aminotransferase and bilirubin levels), but surprisingly, with a higher chance of remission after 12 months of treatment^19^.

In this study, we describe an original, simple strategy to identify the activated TPH and CD8 T cell subsets in the blood of AIH patients by flow cytometry. By comparing the activated TPH frequency, as well as the activated (PD1+CD38+) CD8 T cell frequency, between groups of patients with active AIH, patients in remission and a control group of NASH patients, with liver inflammation, without autoimmunity, we demonstrate that these subsets are associated with the activity of the disease in a wider cohort of patients.

We then investigate whether the frequency of these cell subsets could be of use as potential biomarkers predictive of relapse, in a group of selected patients before initiation of treatment withdrawal. Finally, we also assessed the level of BAFF in the serum of AIH patients and tested its potential association with the risk of relapse.

## Patients and methods

In this retrospective study, all patients with AIH with available frozen Peripheral Blood Monocellular Cells (PBMC) and serum samples in the biobank BIO-MAI-FOIE (CHU Nantes), between 2015 and 2023 were eligible for inclusion. All the patients included had a diagnostic score compatible with AIH according to the simplified scoring system for AIH of the International Autoimmune Hepatitis Group^17^.

Frozen PBMC and serum samples were obtained from the biobank BIO-MAI-FOIE maintained in Nantes University Hospital which is registered as part of the Scientific Research Programs declared to the Ministry of Research by CHU Nantes under number CODECOH DC2017-2987. All the eligible patients signed the informed consent form of CHU Nantes for biobanking which has been approved by the Ethical committee “CPP Ouest IV”. All clinical data were retrieved from electronic patient files. Research performed on these human samples has been performed in accordance with the relevant guidelines and regulations and the Declaration of Helsinki.

Active AIH was defined as new onset AIH patients enrolled at diagnosis prior any treatment initiation, or as patients under IS treatment who do not normalize aminotransferase (AST and ALT) and/or IgG levels (insufficient response) and/or as patients who relapse.

Remission AIH was defined as complete biochemical response (CBR : normalized serum aminotransferase and IgG levels below the upper limits of normal (ULN)) for at least 2 years. Drug withdrawal was done according to EASL guidelines, after at least 3 years of treatment, and 2 years of CBR. Patients for whom drug withdrawal was attempted had a blood sample taken on the first day of drug dose tapering. Liver biopsy prior to cessation of treatment was recommended, but not mandatory to be included in this study. Drug withdrawal consisted of progressive dose tapering of immunosuppressive treatment over 1 year, with relapse defined as increase of aminotransferase above > 2ULN and/or elevation of IgG levels > ULN, and/or necessity of immunosuppressive drug reintroduction.

Patients with histology-proven NASH (Non-Alcoholic Steatohepatitis) were included as control. Upon histological examination, NASH patients presented liver inflammation without autoimmunity.

### Flow cytometry

**Figure 1:**
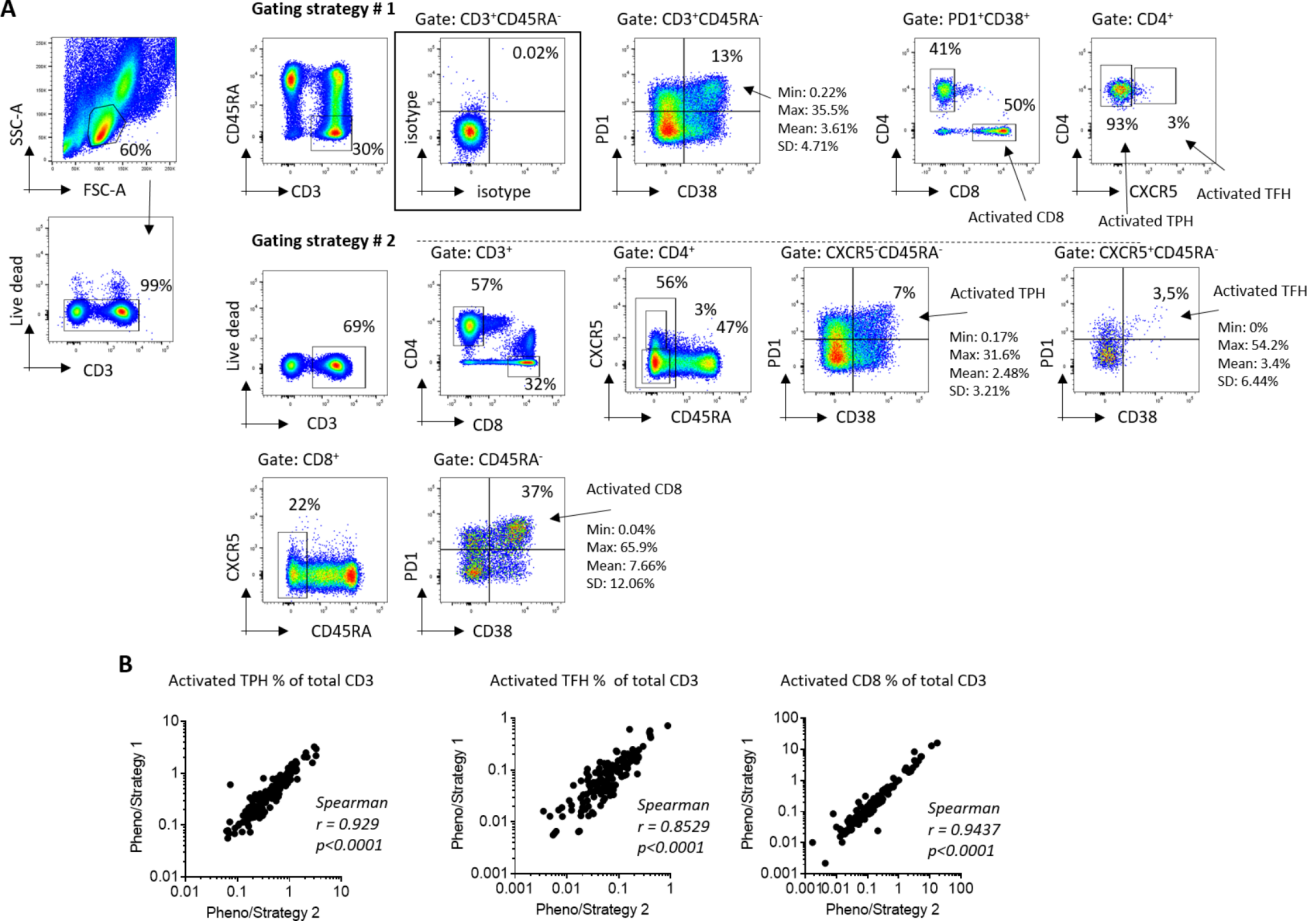
Gating Strategy of total PBMC, to identify and quantify the populations of interest : activated CD8 T cells (CD8+CD45RA-PD1+CD38+) activated TFH cells (CD4+CD45RA-CXCR5+PD1+CD38+); activated TPH cells (CD4+CD45RA-CXCR5-PD1+CD38+). A simple gating strategy #1 consists in gating on memory (CD45RA-) CD3+ T cells, then on CD38+PD1+ T cells to maximize the visibility (percentage between 35 to 0.2%). Isotype controls are used to define the PD1+CD38+ T cells. The fraction of CD4+ and CD8+ T cells is determined on this PD1+CD38+ subset. In the CD4+ fraction, we also isolate T cells based on the CXCR5 expression. A more classical strategy (#2) is shown, which consists in consecutive gating of CD3, then CD4 /CD8, then CXCR5/CD45RA, and finally PD1/CD38. (B) Comparisons of the subset frequencies obtained with two strategies using Spearman correlation test.

PBMC were isolated from total blood samples by Ficoll Plaque gradient centrifugation and stored at -80 °C in the CRB (Centre de Ressources Biologiques) in Nantes University Hospital and INSERM UMR1064. After thawing, PBMC were stained with the following antibodies: CD3 – Alexa Fluor 488, CD4 – PECy7, CD8 – APC-Cy7, CXCR5 – AlexaFluor647, CD45RA - BV421, PD1 – PE and CD38 – PerCp-Cy5.5, all from BioLegend. Flow cytometry was then performed using a BD CANTO II (BD Bioscience). The following T-cell populations were identified with these markers: Total memory (CD45RA-) T cells, CXCR5-PD1+CD38+ CD4 memory T cells (activated TPH), CXCR5+PD1+CD38+ CD4 memory T cells (activated TFH cells) and PD1+CD38+ CD8 memory T cells (activated CD8 T cells). Definitions of these cell populations are shown in Table 1. The gating strategy was performed with FlowJo (TreeStar, Inc.) as detailed in Fig. 1.

**Table 1:** Definitions of T cell populations of interest.

T cell populations (functional description)	Markers
T cell	CD3+
T CD4+	CD3+ CD4+
T CD8+	CD3+ CD8+
T CD4+ memory cell	CD3+ CD4+ CD45RA-
T CD8+ memory cell	CD3+ CD8+ CD45RA-
Activated TFH cell	CD3+ CD4+ CD45RA- CXCR5+ PD1+ CD38+
Activated TPH cells	CD3+ CD4+ CD45RA- CXCR5- PD1+ CD38+
Activated CD8 T cells	CD3+ CD8+ CD45RA- PD1+ CD38+

### ELISA

BAFF levels were determined using DuoSet ELISA (R&D Systems) according to the manufacturer’s protocol. Levels of BAFF were assessed in thawed samples of serum from patients with either active AIH or NASH, and from the patients in remission who underwent treatment withdrawal.

### Statistics

Statistical analysis was performed with GraphPad Prism (version 9.1). Data are presented as mean (Standard deviation) for quantitative variables and count (percentages) for qualitative variables. Nonparametric Mann Whitney test or Kruskal-Wallis test and Dunn’s multiple comparisons test were used to compare cell frequencies. A value of *p* < 0.05 was considered significant. Receiver Operating Characteristic (ROC) Curve Analysis was used to determine significant threshold values, with selection of cut-off value by selecting highest sensibility and specificity. Log Rank survival test was used to determine risk of relapse according do time of follow up. Correlation analysis was performed by Spearman r test.

## Results

### Frequency of activated TPH cells and of activated CD8 T cells are associated with active AIH

We performed a 7-colour flow cytometry on PBMC samples from 58 patients with active AIH, 56 patients with AIH in remission (defined as CBR for at least 2 years), and 31 patients with NASH as a control group. Clinical characteristics of these patients are available in Table 2. Most patients with active AIH were not treated by immunosuppressive drugs at the time of analysis and consisted of a majority of recent diagnostic with no prior treatment (97% of all active AIH). 62% of patients in remission with controlled disease were treated by azathioprine, and only 10 patients (17%) were using steroids at the time of analysis. Seven patients were in remission without treatment. 38% of patients with NASH were Antinuclear Antibodies positive, consistent with the literature^20^.

**Table 2:** Clinical and biological characteristics of patients with AIH and NASH expressed as mean (+/- SD or percentage).

	Active AIH *n* = 58	Remission AIH *n* = 56	NASH *n* = 31
Age (year)	53 ± 19	55 ± 17	59 ± 13
Female, n (%)	43 (72%)	39 (70%)	14 (45%)
IgG (g/L)	22 ± 10	11 ± 2	11 ± 3
AST (IU/L)	480 ± 526	24 ± 7	57 ± 23
ALT (IU/L)	559 ± 607	23 ± 12	89 ± 51
Antibodies at diagnosis n (%) ANA ≥ 1/80 SMA ≥ 1/40 SLA + LKM1+ LC1+ Anti-mitochondrial	45 (77%) 28 (50%) 3 (5%) 1 (2%)	38 (68%) 31 (55%) 5 (9%) 1 (2%) 1 (2%) 2 (3%)	12 (38%) 0 0 0 0 0
Immunosuppressive agents n (%) No, n (%) *Naïve* *Relapse* Yes, n (%) *Azathioprin* *MMF* *Ciclosporin* Steroids only* *Steroids associated to other therapy*	56 (97%) *49* *7*	7 (13%) *-* *-*	31 (100%)
	2 (3%) *1* *-* *1* *-* *2*	49 (87%) *35* *11* *2* *2* *10*	0

*steroids = prednisolone or budesonide

*AIH* Autoimmune Hepatitis, *NASH* Non Alcoholic Steatohepatitis, *AST* Aspartate Aminotransferase, *ALT* Alanine Aminotransferase, *ANA* Antinuclear Antibody, *SMA* Smooth Muscle Antibodies, *SLA* Anti soluble liver antigen, *LKM1* Anti Liver and Kidney Microsome 1, *LC1* Anti Liver Cytosol 1

**Figure 2:**
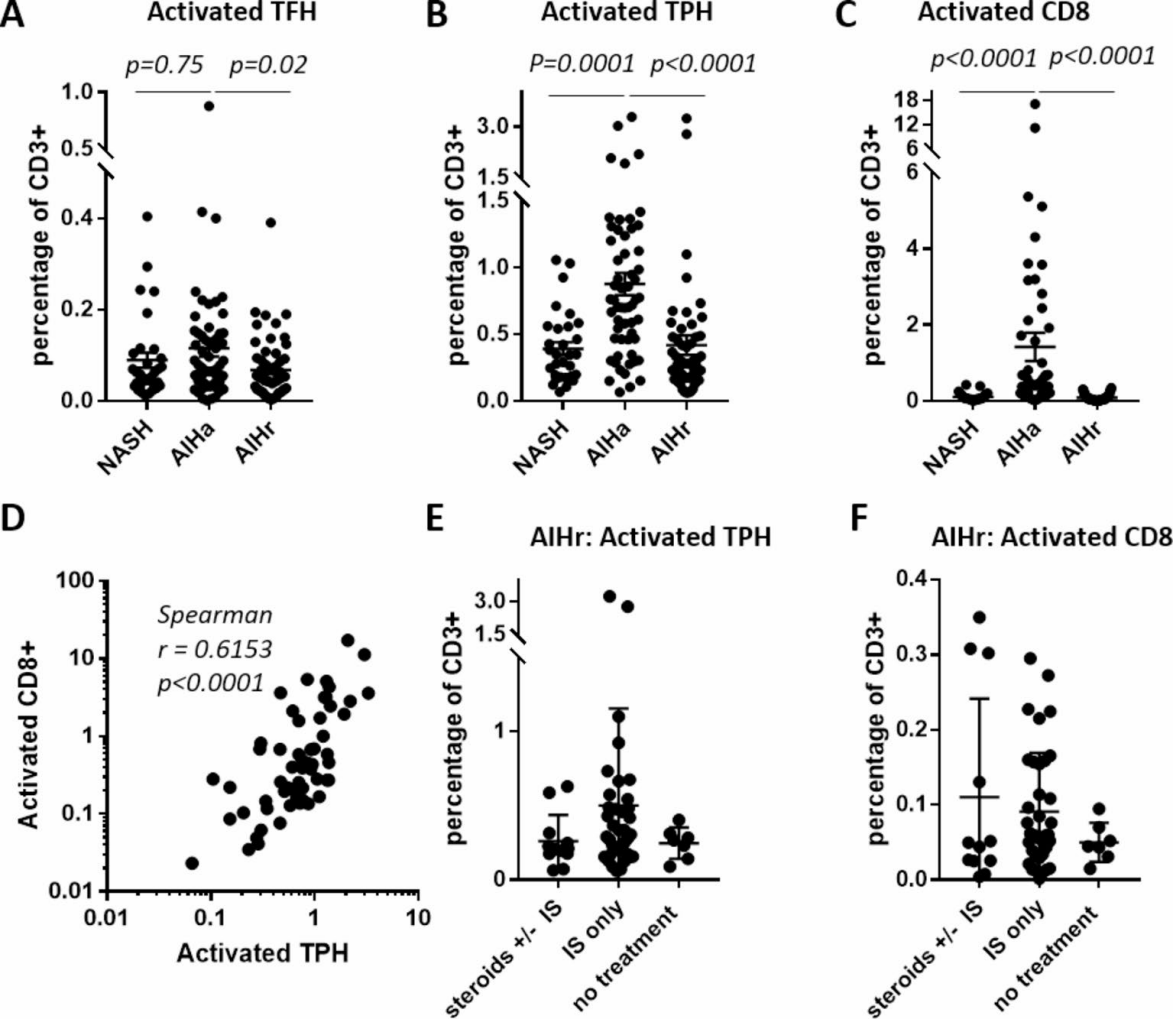
Representative dot plots and graph of the frequency of cell subsets. (**A**) activated TFH cells (CD4+CD45RA-CXCR5+PD1+CD38+); (**B**) activated TPH cells (CD4+CD45RA-CXCR5-PD1+CD38+) and (**C**) activated CD8 T cells, in patients with active AIH (AIHa, *n* = 58), remission AIH (AIHr, *n* = 56) and NASH (*n* = 31), in % of total CD3+ T cells. (**D**) Spearman correlation analysis between the frequencies of activated CD8 T cells and activated TPH cells in patients with active AIH (*n* = 58). Activated TPH (**E**) and CD8 T cells (**F**) in AIH patients in remission, depending on their treatment (Steroids +/- IS *n* = 12, IS only *n* = 37, or no treatment *n* = 7). Kruskal Wallis test and Dunn’s multiple comparisons test were used for A, B, C, E and F.

We used a simple gating strategy (#1) depicted in Fig. 1A, which allowed, after only 4 gating steps to isolate the subsets of interest: activated CD8 T cells (CD3+CD8+ CD45RA-PD1+CD38+), activated TPH (CD3+CD4+CD45RA-CXCR5-PD1+CD38+) and activated TFH (CD3+CD4+CD45RA-CXCR5+PD1+CD38+). This strategy was compared with a classical progressive gating strategy (#2) which required more steps (Fig. 1A). Correlation analyses of the subset frequencies obtained with the 2 strategies, expressed as percent of CD3+ cells showed that they were similar (Fig. 1B). Thus, the simple gating strategy #1 was used for this study to determine the frequency of the 3 subsets of interest in patients with AIH active or in remission, and NASH patients. The frequency of circulating activated TFH cells was not significantly different between active AIH compared to NASH (0.11% of total CD3+ vs. 0.09%, *p* = 0.75), and slightly increased in active AIH compared to AIH in remission (0.11% of total CD3+ vs. 0.07%, *p* = 0.02) (Fig. 2A). Activated TPH cells were found significantly enriched in the blood of patients with active AIH, in comparison to patients with NASH (0.88% of total CD3+ vs. 0.39%, *p* < 0.0001) (Fig. 2B). Frequency of activated TPH cells was also significantly higher in active AIH compared to patients in remission (0.88% of total CD3+ vs. 0.42%, *p* < 0.0001) (Fig. 2B). Similarly, the frequency of activated CD8 T cells was found significantly higher in active AIH compared to NASH and to AIH in remission (1.42% of total CD3+ vs. 0.11% vs. 0.09% respectively, *p* < 0.0001) (Fig. 2C).

These results, consistent with our prior analysis^16^, validate a simplified cytometry panel with only seven markers for the phenotypic tracking of these subsets of interest in a wider cohort. They confirm that active AIH is associated with a higher frequency of activated TPH cells and of activated memory CD8 T cells, but not of activated TFH cells. The frequency of these two activated cellular populations correlate together suggesting a synergic implication in the disease (Fig. 2D).

As mentioned above, treatment varied among patients in remission (Table 2), with some under IS only, or steroids ± IS, and few patients were without treatment (7 out of 56). The frequencies of activated TPH and CD8 T cells were not significantly different between the treatment groups, although AIHr patients without treatment seemed to have lower frequency of these subsets than AIHr under IS treatment (activated TPH: 0.25% vs. 0.50%; activated CD8 0.05 vs. 0.09%) (Fig. 2E and F). This would need further validation on a larger cohort, but it could suggest a link between complete remission and low frequency of PD1+CD38+ T cells.

### Determination of threshold frequency predictive of disease activity

**Figure 3:**
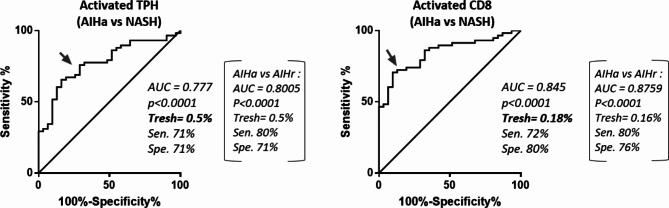
Comparison of frequency of activated T cell subsets between active AIH (AIHa) and NASH, and between AIHa and AIHr (brackets) by AUROC Analysis for determination of significant cut-off values (Tresh). Frequencies are expressed as percent of total CD3+ T cells.

In order to further develop these cell subset frequencies as potential biomarkers, thresholds above which activated TPH cell and activated CD8 T cell frequencies had good predictive value of disease activity were determined, using areas under the receiver operating curves (AUROC) for each cell subset (Fig. 3). The comparison of NASH and active AIH patients in an AUROC analysis led to the determination of 2 values: a threshold of 0.5% of total CD3+ T cells for activated TPH cells, and a threshold of 0.18% of total CD3+ T cells for activated CD8 T cells. Similar analysis to compare AIHr and AIHa patients gave similar results (brackets in Fig. 3). Thus, groups above and underneath each threshold were defined as high/low activated TPH cell group and high/low activated CD8 T cell group, respectively. High level of activated TPH cells was predictive of disease activity with a sensitivity of 71% and a specificity of 71%. High level of activated CD8 T cells was predictive of disease activity with a sensitivity of 72% and a specificity of 80%.

### High frequency of blood activated TPH and/or activated CD8 T cells before drug withdrawal is predictive of relapse

Patients with active AIH have high activated TPH and activated CD8 T cell frequencies, whereas during remission, the frequency of these subsets decreases (Fig. 2). However, some AIH patients in remission displayed persistent high frequency of activated TPH (Fig. 2A). We investigated whether residual higher frequencies of activated TPH and CD8 T cells at the beginning of immunosuppressive drug dose tapering was associated with a higher risk of relapse. Activated TPH cells and activated CD8 T cells were tracked in PBMC samples from 18 patients with controlled AIH who then underwent drug withdrawal. Characteristics of patients are depicted in Table 3. The median time with normal aminotransferases and IgG prior to tapering of immunosuppression was 5 years (range 3–12). Only 4 patients had liver biopsy prior to drug withdrawal. Most patients were receiving azathioprine at the onset of dose tapering. Three patients were treated by mycophenolate mofetil due to intolerance to azathioprine. Relapse occurred in 33% of patients within the two years following treatment withdrawal. The direct comparison of AST, ALT and IgG level did not seem to be linked to relapse. However, frequency of activated TPH and of activated CD8 T cells was high in the relapse group before treatment withdrawal, but did not reach statistical significance.

**Table 3:** Clinical characteristics of patients undergoing treatment withdrawal (*n* = 18) based on relapse or no relapse (mean +/- SD; Mann-Whitney test).

	Relapse	No relapse	*P* value
n	6	12	
Female, n (%)	4 (67%)	7 (58%)	ns
Age (year)	61.2 ± 16.1	45.3 ± 19.5	0.1186
Age at diagnosis (year)	52.5 ± 13.5	38.8 ± 18.2	0.1718
Median duration of disease, year (range)	8 (4–16)	5.5 (3–13)	0.4183
Treatment stopped, n (%) Azathioprin Mycophenolate mofetil	5 (83%) 1 (17%)	10 (83%) 2 (17%)	
AST (UI/l)	22.1 ± 6.5	20.4 ± 4.9	0.3724
ALT (UI/l)	22.2 ± 10.5	18.1 ± 12.3	0.1441
IgG (g/l)	11.3 ± 1.8	10.6 ± 2.2	0.6605
Activated TPH per CD3 (%)	0.85 ± 0.97	0.27 ± 0.14	0.0668
Activated CD8 per CD3 (%)	0.15 ± 0.10	0.07 ± 0.07	0.0831
BAFF (pg/ml)	268.8 ± 99.08	185.8 ± 61,95	0.0897

**Figure 4:**
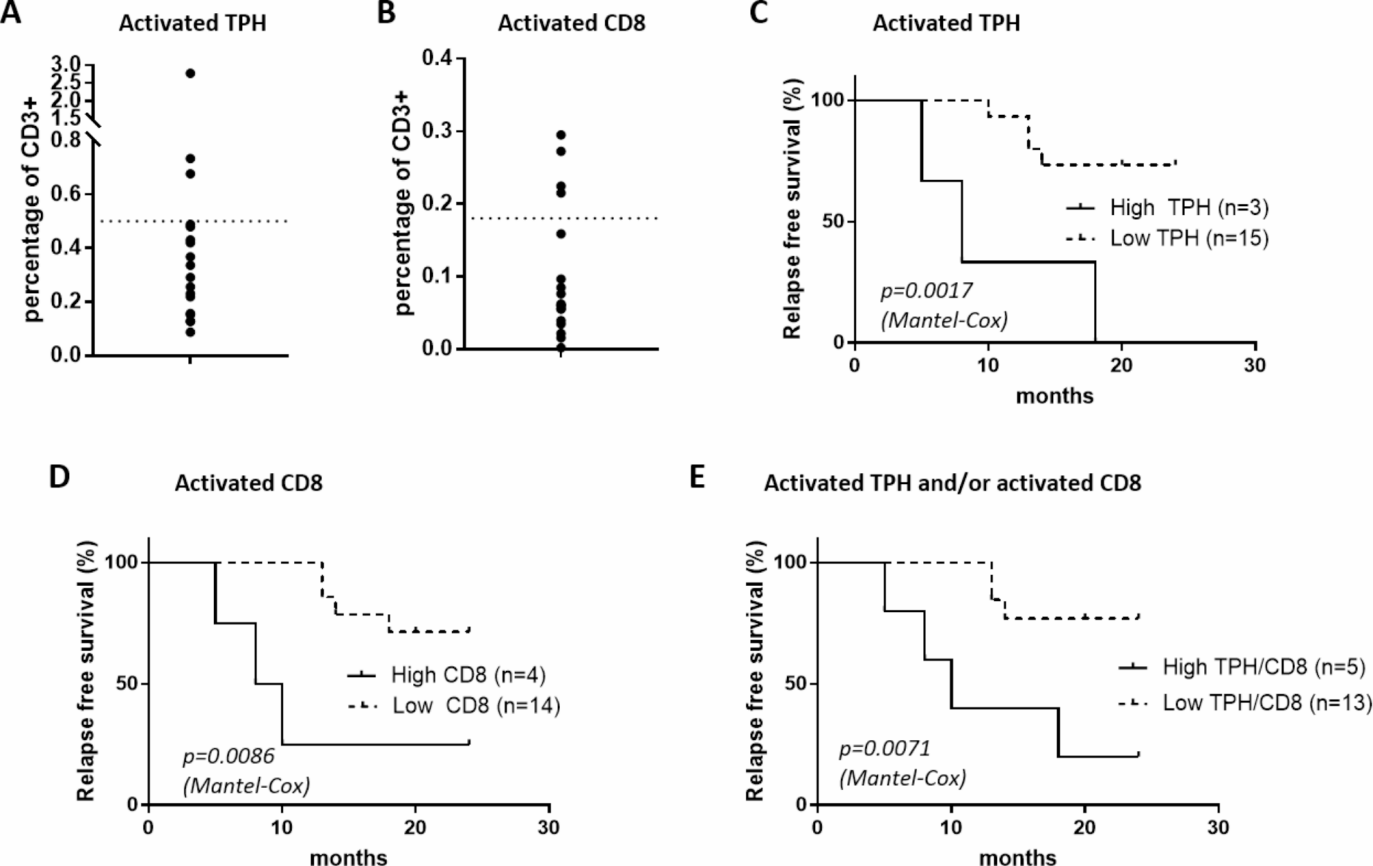
Graph representation of the frequency of activated TPH cells (**A**) and activated CD8 T cells (**B**), in the cohort of AIH patients in remission before treatment withdrawal (*n* = 18). Dotted lines represent the thresholds determined in Fig. 3. (C - E) Relapse-free survival stratified by the frequency of activated TPH (group high TPH, *n* = 3; group low TPH, *n* = 15) (**C**) or of activated CD8 T cells (group high CD8, *n* = 4; group low CD8, *n* = 14) (**D**), or combined frequencies of activated TPH and/or of CD8 T cells group high TPH/CD8, *n* = 5; group low TPH/CD8, *n* = 13) (**E**), determined by flow cytometry. Log-rank (Mantel Cox) analysis.

Patients undergoing treatment withdrawal were categorized based on previously established thresholds of 0.5% of total CD3+ T cells for activated TPH cells and 0.18% of total CD3+ T cells for activated CD8 T cells. This categorization aimed to assess the impact of these cell frequencies on relapse during the initial two years post-treatment withdrawal (Fig. 4A and B). Patients in the high activated TPH cells group had a significantly increased risk of relapse (Fig. 4C; *p* = 0.0017). Patients in the high activated CD8 T cells group also had a significantly increased risk of relapse (Fig. 4D; *p* = 0.0086). By combining the two frequencies, high activated TPH and/or high activated CD8 T cells, patients had a significantly increased risk of relapse (Fig. 4E; *p* = 0.0071). The risk of relapse at 2 years of follow up reached 80% (*n* = 4 out of 5) in the high-group vs. 15% (*n* = 2 out of 13) in the low-group.

### Association of serum concentration of BAFF with AIH activity and risk of relapse

**Figure 5:**
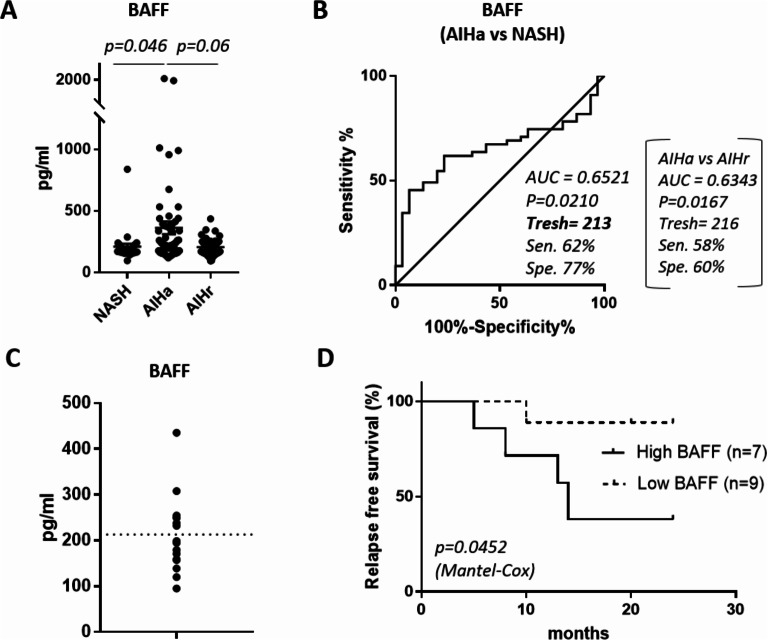
BAFF levels and risk of relapse. (**A**) Quantification of BAFF levels in NASH patients (*n* = 30) and active AIH (AIHa, *n* = 55), AIH in remission (AIHr, *n* = 52). (**B**) ROC analysis of BAFF levels for AIHa vs. NASH (graph), and AIHa vs. AIHr (brackets). (**C**) Quantification of the level of BAFF in AIH patients in remission before treatment withdrawal (*n* = 16), a dotted line represents the threshold determined in B. (**D**) Relapse-free survival, stratified by the serum level of BAFF measured in AIH patients in remission before treatment withdrawal (group high BAFF, *n* = 7; group low BAFF, *n* = 9).

To investigate if high level of BAFF could also be a biomarker of interest, we first measured BAFF concentration in the serum of 55 AIHa, 52 AIHr, and in 30 NASH patients. BAFF levels were significantly higher in the serum of active AIH than in NASH patients (362.4 vs. 210.7pg/ml, *p* = 0.046) (Fig. 5A). The comparison of these two groups of patients in an AUROC analysis led to the setting of a threshold of BAFF concentration at 213 pg/ml (Fig. 5B). Similar results were obtained when comparing AIHa to AIHr groups (Fig. 5A and B (brackets)).

The impact of BAFF levels on the risk of relapse was tested on a group of 16 patients undergoing treatment withdrawal. BAFF levels in patients who relapsed and in those who didn’t were not significantly different (268.8 vs. 185.8 pg/ml *p* = 0.089, Table 3).

Patients were stratified into 2 groups according to high (≥ 213pg/ml, *n* = 7) or low BAFF (< 213pg/ml, *n* = 9) at the start of IS dose tapering (Fig. 5C). The high BAFF group had a higher risk of relapse (high BAFF 57% of relapse vs. low BAFF 11% of relapse at 2 years of follow-up, *p* = 0.0452) (Fig. 5D).

### Flow cytometry analysis on fresh whole blood sample

**Figure 6:**
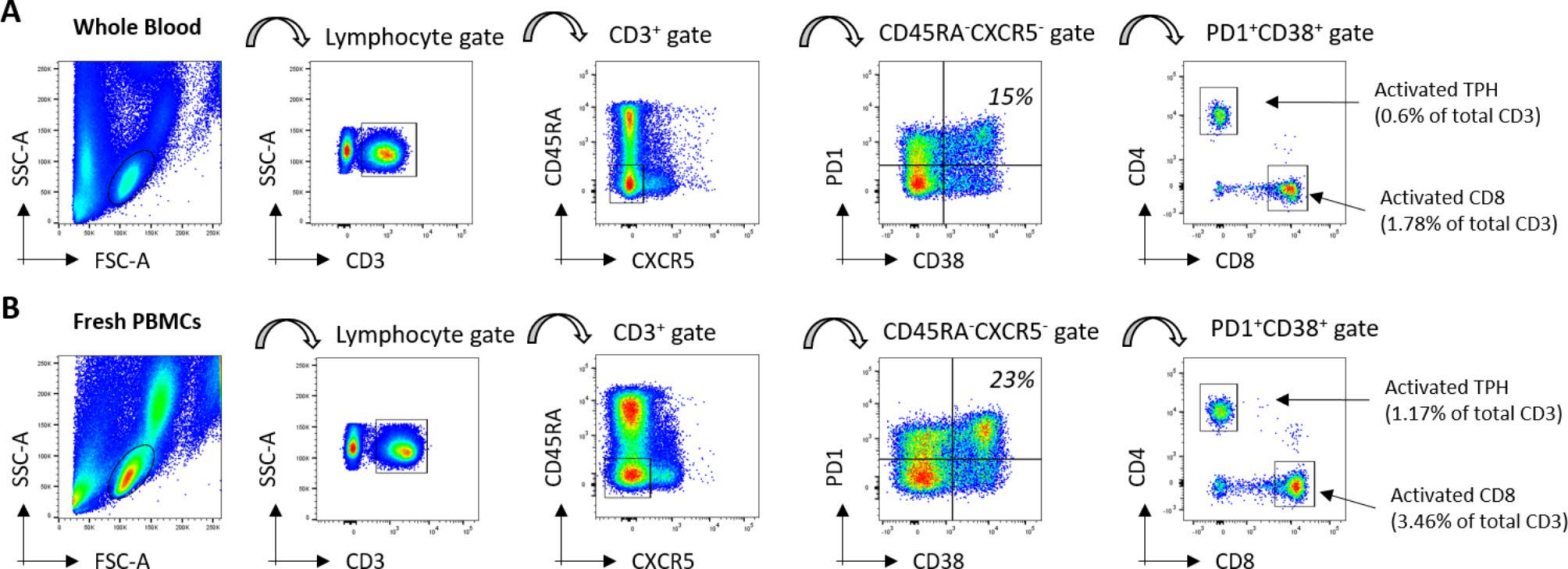
Flow cytometry analysis on fresh whole blood and isolated PBMC. (**A**) Gating strategy of whole blood sample. (**B**) gating strategy on freshly isolated PBMC from the same AIHa patient.

In order to propose a simple clinical test, we performed the same flow cytometry experiment on a blood sample from an active AIH patient. We compared the results obtained with a limited amount (200 µl) of fresh whole blood and with PBMC isolated from 5 ml of blood using a Ficoll gradient (Fig. 6A and B). In both cases, we were able to stain T cells (CD3+), then we gated the CD45RA-CXCR5- cells (exclusion of the TFH cells) and then we focused on PD1+CD38+ T cells to determine the frequency of activated TPH cells (CD3+CD45RA-CXCR5-PD1+CD38+CD4+) and of activated CD8 cells (CD3+CD45RA-CXCR5-PD1+CD38+CD8+) per total CD3 was calculated. Although, differences were observed between these two protocols, the values were in the range measured for frozen samples of active AIH patients (Fig. 2B and C). Thus, this test could be easily transferred into clinical practice.

## Discussion

The actual course of autoimmune hepatitis is marked by a high risk of relapse upon attempting IS treatment withdrawal. Despite this acknowledged risk, International guidelines recommend considering treatment cessation if possible^21^. Almost all patients want to try stopping treatment. These attempts require that strict monitoring criteria are respected and that patients have a good understanding of the issues. They are part of the therapeutic alliance, the permanent dialogue between doctors and patients. Some clinical features have been associated to higher risk of relapse (anti SLA antibodies, higher values of aminotransferase in the normal range, young age)^5,22^, however they still need further validation. Liver biopsy remains recommended (but not mandatory) to verify that the patient is in remission, but the acceptability of this procedure is low. The development of non-invasive biomarkers enabling risk stratification of patients prior to the gradual reduction of treatment would facilitate clinical decision-making regarding the timing of withdrawal and the frequency of monitoring after withdrawal.

In this study, we investigated the potential use of circulating activated TPH cells and activated CD8 T cells, both expressing PD1 and CD38, to look for residual disease activity of AIH and predict relapse after IS treatment withdrawal.

Previously, based on a multiparametric flow cytometry study, we uncovered an association between the frequency of circulating activated TPH and CD8 T cells and the disease activity. This work was however based on a complex gating strategy designed to discover new circulating T cell populations, on a limited number of patients. One important quality criterion for a potential biomarker is its practical implementation in the clinical environment. Thus, one objective of this work was to simplify the tracking of these populations, while validating the statistical significance of the association with the activity of the disease. We validated a straightforward method based on a seven-color flow cytometry strategy, on a larger number of patients, confirming the robustness of the association. Although the frequency of the populations of interest is small, we show that they can be reliably detected and quantified on frozen PBMC, but also on fresh PBMC and on fresh whole blood samples, a pre requisite to further develop a clinical test.

In the present study, the frequency of two populations of circulating T cells, activated TPH and activated CD8 T cells are significantly higher in the blood of patients with an active AIH compared to AIH patients in remission, and to patients with NASH, taken as a control group presenting liver inflammation, without autoimmunity. This choice of control group was dictated by previous results on circulating T cell subset comparisons between AIH and healthy volunteers, where some variations were attributable to the background liver inflammation rather that to the specific autoimmune events of AIH^23^.

The detailed analysis of the group of AIH patients in remission revealed a certain heterogeneity with some of them with higher activated TPH and CD8 T cells frequencies despite at least 2 years of biochemical remission. We hypothesized that these could reveal remnant activity despite apparent disease control and could therefore expose patients to higher risk of relapse.

This study confirms how high activated TPH and CD8 T cells are strongly associated to risk of relapse, and how quantification of these cell subsets could be a useful clinical tool to guide decision of treatment withdrawal.

There are increasing reports in the literature on the role of TPH cells in autoimmune diseases^12,24,25^. Autoimmune hepatitis is no exception and TPH cells seem to play an important role in the disease. Enriched in peripheral blood of patients with active autoimmune hepatitis, they could promote B-differentiation and potentially autoreactive CD8+ T cell cytotoxicity and, to our knowledge AIH is the first autoimmune disease for which the frequency of these circulating cells is demonstrated to have a predictive value and could be used as a biomarker to risk stratify patients prior to treatment withdrawal attempt.

Elevated BAFF levels have been described in the serum of AIH patients^26^, and seem to be linked to the severity of the disease. Therapeutically, BAFF inhibitor belimumab has been shown to be effective in other autoimmune diseases (e.g. systemic Lupus Erythematous, SLE^27^), and recently case reports suggest it could be a valid third line treatment for refractory AIH patients^28,29^. The potential use of BAFF levels to identify different immunological phenotypes of AIH, with different presentation, treatment response and outcome has been studied. Patients with high serum level of BAFF at baseline presented higher bilirubin than patients with normal level of BAFF. In the same study, the decrease of BAFF level under immunosuppressive treatment was associated to decrease of number of plasma cells evaluated by flow cytometry^19^.

In our study, we hypothesized that if BAFF levels are associated with the severity of the disease, high levels of BAFF in patients in remission at starting point of treatment withdrawal could predict a higher risk of relapse. Indeed, we observe a high risk of relapse in the high BAFF compared to the low BAFF group (57% vs. 11% at 24 months).

Finally, the correlation observed between the frequencies of activated TPH cells and activated CD8 T cells, could suggest a dual upstream role of CD4 TPH cells in AIH that influences both T and B cell responses, thus contributing to the pathogenesis of AIH.

However, this study has its limitations, as the number of patients studied prior to treatment discontinuation is small. Moreover, the number of patients with a high frequency of activated lymphocytes (TPH and CD8) prior to treatment discontinuation was limited (*n* = 5). This study is therefore exploratory, and other clinical studies will have to be carried out on larger cohorts. Our study supports the idea that it would be relevant to include the study of blood lymphocyte populations in future prospective clinical studies to determine the risk of relapse before treatment discontinuation. Detailed knowledge of immune cell subsets is essential for the diagnosis and the personalized clinical follow-up of patients with autoimmune diseases. This study gives new perspectives into the use of activated TPH, activated CD8 T cells and BAFF levels as clinical parameters, and their potential use as biomarkers of the risk of relapse in AIH patients.

## Data Availability

All data generated or analysed during this study are included in this article.
